# Exercise as a promising alternative for sciatic nerve injury pain relief: a meta-analysis

**DOI:** 10.3389/fneur.2024.1424050

**Published:** 2024-07-31

**Authors:** Shunxin Liu, Qin Li, Huaiming Wang, Hongwei Zhang, Qi Zhao, Jinjun Su, Jiang Zou, Pengjiu Feng, Aimin Zhang

**Affiliations:** ^1^Guangxi University of Chinese Medicine, Nanning, China; ^2^Department of Anesthesiology, Sichuan Clinical Research Center for Cancer, Sichuan Cancer Hospital and Institute, Sichuan Cancer Center, Affiliated Cancer Hospital of University of Electronic Science and Technology of China, Chengdu, China; ^3^Department of Anesthesiology, Liuzhou Traditional Chinese Medicine Hospital, The Third Affiliated Hospital of Guangxi University of Chinese Medicine, Liuzhou, China

**Keywords:** pain model, exercise, sciatic nerve injury, neuropathic pain, meta-analysis

## Abstract

**Objective:**

The efficacy of drug therapies in managing neuropathic pain is constrained by their limited effectiveness and potential for adverse effects. In contrast, exercise has emerged as a promising alternative for pain relief. In this study, we conducted a systematic evaluation of the therapeutic impact of exercise on neuropathic pain resulting from sciatic nerve injury in rodent models.

**Methods:**

The PubMed, Embase, and Web of Science databases were retrieved before April 2024. A series of studies regarding the effect of treadmill, swimming, wheel and other exercises on neuropathic pain induced by sciatic nerve injury in rats and mice were collected. Using predefined inclusion criteria, two researchers independently performed literature screening, data extraction, and methodological quality assessment utilizing SYRCLE’s risk of bias tool for animal studies. Statistical analysis was conducted using RevMan 5.3 and STATA 12.0 analysis software.

**Results:**

A total of 12 relevant academic sources were included in the analysis of controlled animal studies, with 133 rodents in the exercise group and 135 rodents in the sedentary group. The meta-analysis revealed that exercise was associated with a significant increase in paw withdrawal mechanical threshold [Standard Mean Difference^1^ (*SMD*) = 0.84, 95% confidence interval (*CI*): 0.28–1.40, *p* = 0.003] and paw withdrawal thermal latency (*SMD* = 1.54, 95%*CI*: 0.93–2.15, *p* < 0.0001) in rats and mice with sciatic nerve injury. Subgroup analyses were conducted to evaluate the impact of exercise duration on heterogeneity. The results showed that postoperative exercise duration ≤3 weeks could significantly elevate paw withdrawal mechanical threshold (SMD = 1.04, 95% *CI*: 0.62–1.46, *p* < 0.00001). Postoperative exercise duration ≤4 weeks could significantly improve paw withdrawal thermal latency (SMD = 1.93, 95% *CI*:1.19–2.67, *p* < 0.00001).

**Conclusion:**

Exercise represents an effective method for improving mechanical and thermal hypersensitivity resulting from sciatic nerve injury in rodents. Factors such as pain models, the initiation of exercise, the type of exercise, and the species of rodent do not significantly impact the development of exercise-induced hypoalgesia. However, the duration of postoperative exercise plays a crucial role in the onset of exercise-induced hypoalgesia.

## 1 Introduction

Chronic pain is a pervasive global issue that incurs substantial treatment expenses and imposes significant burdens on both society and families (1). Additionally, individuals suffering from chronic pain frequently experience comorbid anxiety and depression (2).

Symptoms of chronic pain, particularly insomnia, can result in considerable physical and psychological suffering (3, 4). Chronic pain frequently manifests with a neuropathic component, affecting an estimated 10% of cases (5) and is characterized by intricate pathophysiological mechanisms involving a complex interplay of neurotransmitters, receptors, ion channels, and cellular processes. The intricate nature of these pain factors presents a significant challenge in developing effective treatment strategies (6).

Pharmacological interventions represent a mainstay of managing pathological pain, despite the potential side effects of nausea, drowsiness, weight gain, and pruritus (7). However, the efficacy of medication alone is often limited, necessitating the incorporation of non-pharmacological treatments such as surgery, electrical stimulation, stem cell therapy, acupuncture, and exercise to achieve optimal therapeutic outcomes (8). These complementary approaches have demonstrated positive therapeutic effects in the management of pathological pain (9, 10).

Exercise, as a non-pharmacological intervention, has shown significant effects in pain management. It mainly reduces pain through several mechanisms. First, exercise promotes blood circulation, accelerates tissue repair and inflammation resolution, which is the key to relieving acute and chronic pain. At the same time, it enhances muscle strength and flexibility, improves body posture, and reduces pain caused by poor posture or muscle tension. Secondly, exercise’s positive regulation of the nervous system is also an important way to relieve pain. It can stimulate the endogenous analgesic system, release natural analgesic substances such as endorphins, regulate the transmission of pain signals, and reduce pain perception. In addition, exercise promotes neuroplasticity, including neuron regeneration and synaptic reconstruction, which helps restore damaged nerve function and relieve neuropathic pain. Exercise-induced pain relief has become a research hotspot in recent years due to its intricate link to the physiological responses of the human body. As a pain management modality, exercise offers a unique advantage in its ability to exert its influence on multiple organs and systems simultaneously, thereby benefiting the treatment of diverse medical conditions (11). Several studies have indicated that inadequate physical activity may contribute to the exacerbation of pain. Research conducted on both human subjects and rats has demonstrated that limitations in physical activity can result in heightened sensitivity to pain (12, 13).

Understanding the impact and underlying mechanisms of pain significantly influences clinical decision-making, providing a theoretical basis for the development of effective exercise intervention programs (14, 15). Meta-analyses on the effectiveness of exercise for pain relief are inconclusive due to substantial variations in pain models and measurement methods. These variations may also explain inconsistencies in research findings related to exercise methods, styles, intensity, duration, and specific pain conditions (16–18).

While numerous studies support the effectiveness of exercise for pain relief, others suggest it may not always be beneficial (19). Consequently, it is imperative to investigate the impact of various exercise regimens on distinct types of pain to prescribe exercise interventions that yield optimal results.

This study employs meta-analysis techniques to comprehensively evaluate the efficacy of diverse exercise interventions for alleviating pain in animal models of sciatic nerve injury. The primary objective is to elucidate the impact of exercise on the functional restoration of sciatic nerve tissue. Ultimately, this investigation aims to inform the development of future research on exercise prescription for pain management strategies. By exploring the potential of exercise-based interventions, this research contributes to the growing body of knowledge on multimodal treatment approaches for neuropathic pain, potentially complementing pharmacological interventions and stem cell therapies for a more comprehensive therapeutic approach.

## 2 Data and methods

The article adhered rigorously to the Preferred Reporting Items for Systematic Reviews and Meta-analyses (PRISMA) guidelines (20).

### 2.1 Literature search strategy

**Figure 1:**
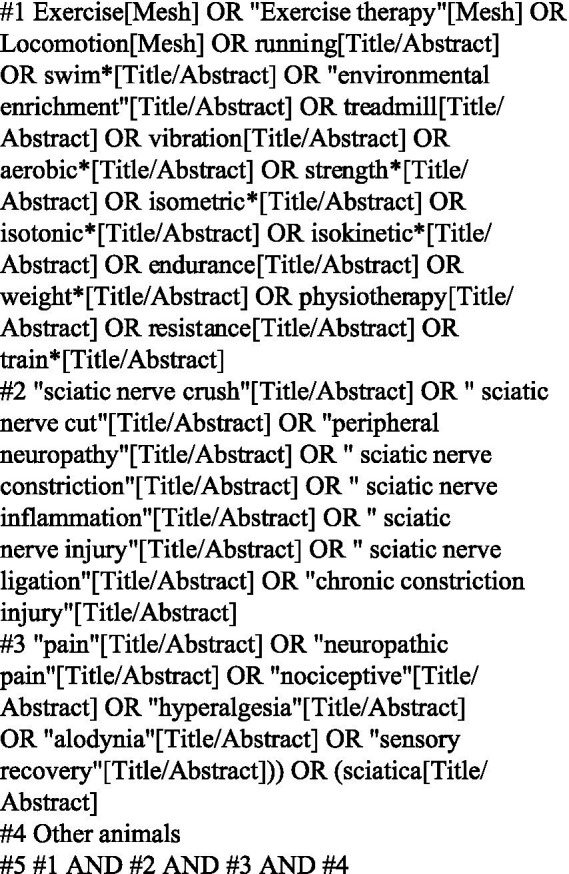
Retrieval strategies for PubMed database.

A comprehensive literature search strategy was employed to identify relevant studies for this systematic review. The search was conducted by the author across four electronic databases: Cochrane Library, PubMed, Embase, and Web of Science. To ensure thoroughness, a broad range of search terms were used, encompassing various exercise modalities (exercise, locomotion, running, swimming, environmental enrichment, treadmill, vibration, aerobic, strength, isometric, isotonic, isokinetic, endurance, weight, physiotherapy, resistance, training), alongside terms related to sciatic nerve injury (sciatic nerve crush, nerve ligation, chronic constriction injury) and pain (pain, neuropathic pain, nociceptive, hyperalgesia, alodynia, sensory recovery, sciatica). Notably, the search encompassed studies published from December 2023 onwards. The specific search strategy employed within each database is detailed elsewhere (potentially in Figure 1). In addition to the electronic search, manual inspection of retrieved articles and reference lists was conducted to ensure the capture of potentially relevant studies not identified by the initial database search. This comprehensive approach aimed to maximize the identification of relevant studies for inclusion in the systematic review.

### 2.2 Inclusion and exclusion criteria

#### 2.2.1 Inclusion criteria

1. Research object: Basic experimental research on the construction of a sciatic nerve injury pain model in rats and mice.
2. Exercise Intervention: All animals in the study underwent a postoperative exercise intervention.
3. Control Group: A control group was established that included animals with the sciatic nerve injury pain model but without exercise intervention.
4. Outcome Measures: Studies had to report at least one of the following pain outcome measures: (1) Mechanical pain threshold measured by von Frey filaments; (2) Thermal pain threshold measured by thermal radiation.
5. Study Design: Controlled animal experiments were included.

#### 2.2.2 Exclusion criteria

1. Absence of a Control Group.
2. Duplicate Studies: Studies with redundant data were excluded, such as those from the same author using the same experimental subjects and procedures.
3. Insufficient Data.
4. Animals with Altered Pain Perception.
5. Review Articles.

#### 2.2.3 Literature screening and data extraction

The author independently conducted literature screening, data extraction, and cross-checking to ensure accuracy and minimize bias. In cases of disagreement, a third party was consulted to reach a consensus. If essential information was missing from the studies, attempts were made to contact the original authors for clarification. Additionally, Engage Digitizer software was utilized to extract data from measurement charts where necessary. The extracted data encompassed the following categories:

1. Basic Study Information: This included the first author, country/region of publication, and publication year.
2. Subject Characteristics: This included animal species, age, body mass, sex, and the specific pain model used.
3. Intervention Details: This included the duration, mode, intensity, and start time of exercise interventions for the experimental group, as well as any interventions administered to the control group.
4. Risk of Bias Assessment: Key elements were evaluated to assess the potential risk of bias within the included studies.
5. Outcome Measures: This included the specific pain outcome indicators employed in the studies, along with the corresponding data.

#### 2.2.4 Literature quality assessment

The risk of bias in the original animal experiments included in this study was assessed using the Systematic Review Centre for Laboratory Animal Experimentation (SYRCLE) animal experiment risk of bias assessment tool recommended by SYRF (21). The evaluation was conducted independently by the author, who reviewed the included articles for research bias across various aspects:

1. Sequence generation.
2. Baseline characteristics.
3. Allocation concealment.
4. Animal placement: This refers to whether the randomization process included assigning animals to different cages or housing conditions to minimize bias.
5. Blinding of Researchers.
6. Randomized outcome evaluation.
7. Blinding of testers.
8. Incomplete data reporting.
9. Selective Outcome Reporting.
10. Other sources of bias.
11. For each criterion, “Y” indicated a low risk of bias, “N” indicated a high risk of bias, and “U” indicated unclear risk.

### 2.3 Outcome

The specific outcomes assessed in this study were mechanical pain threshold and changes in thermal pain threshold.

### 2.4 Statistical analysis

Data analysis was conducted using RevMan 5.3 and STATA 12.0, provided by the Cochrane Library Collaboration Network, with software from StataCorp in College Station, Texas, United States. The heterogeneity of the included studies was assessed using the *I^2^* statistic. A value of *I*^2^ < 50% and a Q-test *p*-value > 0.1 indicated low heterogeneity, allowing for the use of a fixed-effects model for analysis. Conversely, *I*^2^ ≥ 50% or a Q-test *p*-value ≤ 0.1 indicated significant heterogeneity, necessitating the use of a random-effects model. Since the outcome indicators (mechanical and thermal pain thresholds) were continuous data, the standardized mean difference (SMD) with its corresponding 95% confidence interval (*CI*) was used for analysis. Based on the analysis results, subgroup and sensitivity analyses were conducted to explore potential sources of heterogeneity. Additionally, Egger’s test was employed for quantitative assessment of publication bias. A *p*-value < 0.05 from Egger’s test indicated potential publication bias. In such cases, the trim and fill method was utilized to potentially adjust for this bias.

## 3 Results

### 3.1 Literature retrieval

**Figure 2:**
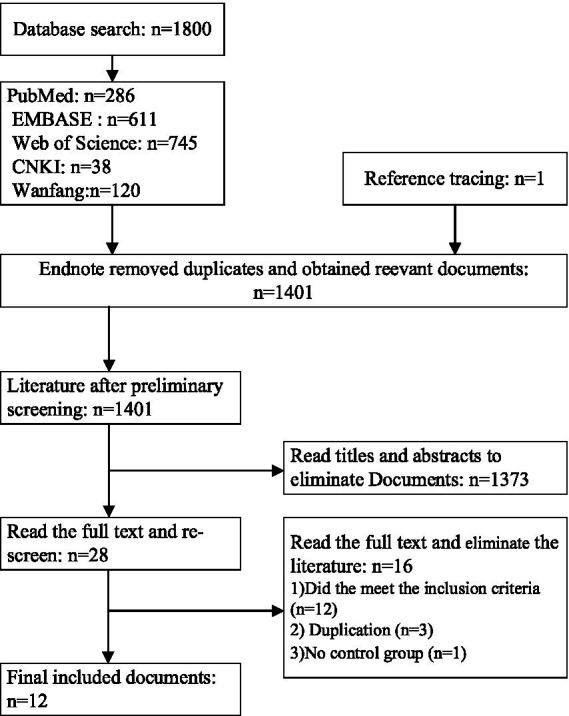
Flowchart of the literature retrieval process.

Following the outlined literature retrieval strategy and systematic search of library databases for relevant scholarly articles, a total of 1,801 articles were identified (Figure 2). The breakdown by database was as follows: PubMed (*n* = 286), EMBASE (*n* = 611), Web of Science (*n* = 745), CNKI (*n* = 38), and Wanfang (*n* = 120). Utilizing Endnote software, duplicate articles (*n* = 400) were removed. Title and abstract screening excluded an additional 1,373 articles. Finally, full-text review of the remaining articles resulted in the exclusion of 16, yielding a final selection of 12 studies for inclusion in this analysis.

### 3.2 Basic characteristics of studies included in this research

This meta-analysis analyzed twelve controlled animal experiment articles published prior to April 2024 that explored the impact of exercise on pain management (16–18, 22–30). Three of these articles included two separate exercise regimens (16, 22, 29), resulting in a total of fifteen exercise protocols evaluated. The study population consisted of both rats (68.9%) and mice (31.1%). Within the rat population, 133 animals were assigned to the exercise intervention group, with an equal number assigned to the control group, for a total of 266 animals (133 rats and 133 mice).

The studies employed various sciatic nerve injury models, including partial ligation, chronic compression, and resection-repair. Within the exercise programs, treadmill exercise was most frequent (9 studies) (16, 18, 22, 23, 25–27). Swimming exercise programs were used in four studies (17, 22, 24, 29), and one study utilized a voluntary wheel running (28). Five studies initiated exercise interventions before surgery (18, 22, 27–29), while seven began exercise post-surgery (16, 17, 23–26, 30). Postoperative exercise duration ranged from 1 to 8 weeks with no reported adverse events (18, 23). Both control and exercise groups received the same pain model induction, with the control group not receiving any exercise intervention. This study focused on the therapeutic effects of exercise on mechanical and thermal hyperalgesia due to limited research on cold allodynia and exercise-induced spontaneous pain.

To ensure good homogeneity among studies, the included outcome indicators were the mechanical pain threshold measured by Von Fery filaments and the thermal pain threshold measured by thermal radiation. The comparisons based on different models are shown in Tables 1A–C.

**Table 1:** Characteristics of the included studies with **(A)** PSNL model, **(B)** CCI model, and **(C)** SNTR model.

Author/year of publication	Animal species/sex/body weight	Pain model	Sample size (exercise group/control group, *n*)	Movement begin time	Exercise interventions	Outcome measures
**(A) PSNL model**						
Almeida et al. (17), 2015	BALB/c mouse, male (23.5 + 0.24) g	PSNL	10/12	7 days postoperatively	Swimming, 1 min/d, preoperative acclimatization for 5 d. Start with 10 min/d and increase every 3 days of training 10 min, to 50 min, then stop increasing, 5/week, for a total of 5 weeks	Hot pain threshold
Kami et al. (27), 2016	C57BL/6 mouse, male, Weight was not provided	PSNL	6/6	12 days preoperatively	Treadmill, 10 min/d 2 weeks before surgery, 20–60 min 1 week before surgery, 2 days after surgery, 60 min/d for 5 consecutive days, 7 m/min	Hot pain threshold
Kami et al. (28), 2018	C57BL/6 J mouse, Weight was not provided	PSNL	8/6	14 days preoperatively	Runner, 14 days preoperatively to 14 days postoperatively	Hot pain threshold
Kuphal et al. (29), 2007	Sprague–Dawley rat, male, 250–300 g	PSNL	6/8	14 days preoperatively	Swimming, 14 days before surgery to 25 days after surgery, 90 min/d	Hot pain threshold
Kuphal et al. (29), 2007	CD1 mouse, male, 30–35 g	PSNL	10/10	5 days preoperatively	Swimming, continuous exercise for 5 days before surgery, and continuous exercise for 6 days after 1 day after surgery, 30 min/d	Hot pain threshold
**(B) CCI model**						
Chen et al. (22), 2012	Sprague–Dawley rat, male, 250–300 g	CCI	10/10	2 days preoperatively	Treadmill, no slope, from 20 m/min to 30 m/min, 15–60 min/d, 5 d/week, 6 weeks in total	Mechanical pain threshold, Hot pain threshold
Chen et al. (22), 2012	Sprague–Dawley rat, male, 250–300 g	CCI	10/10	2 days preoperatively	Swimming, start with 10 min/repetition and gradually increase to 90 min/repetition 1 training session per day for 90 min for 39 days	Mechanical pain threshold, Hot pain threshold
Cobianchi et al. (18), 2010	CD1 mouse, male, 40–45 g	CCI	8/11	14 days preoperatively	Treadmill, 12 m/min start, 1.2–31.2 m/min increments every 5 min, 1 h/d, 5 days/week, 2 weeks preoperatively plus 3–56 days postoperatively	Mechanical pain threshold
Huang et al. (25), 2017	Sprague–Dawley rat, male, 220–270 g	CCI	10/10	8 days postoperatively	Treadmill, 8% uphill, 14–16 m/min, 30 min/d, 3 weeks	Mechanical pain threshold, Hot pain threshold
Hung et al. (26), 2016	Sprague–Dawley rat, male, 220–270 g	CCI	10/10	3 days postoperatively	Treadmill, 8% uphill, 14–16 m/min, 30 min/d, 5 d/week for 4 weeks	Mechanical pain threshold, Hot pain threshold
Tsai et al. (16), 2017	Sprague–Dawley rat, male, 285–335 g	CCI	12/12	3 days postoperatively	Treadmill, 14–16 m/min, 30 min/d, 8% uphill, 3 weeks of continuous movement	Mechanical pain threshold, Hot pain threshold
Tsai et al. (16), 2017	Sprague–Dawley rat, male, 285–335 g	CCI	12/12	2 days postoperatively	Treadmill, 14–16 m/min, 30 min/d, no slope, 3 weeks of continuous movement	Mechanical pain threshold, Hot pain threshold
Farzad et al. (24), 2018	Wistar rat, male, 180–220 g	CCI	7/7	3 days postoperatively	Swimming, 3 days of acclimatization training, the exercise time gradually increased to 60 min/d in the first week, and the total movement was carried out Move for 4 weeks	Mechanical pain threshold, Hot pain threshold
Safakhah et al. (30), 2017	Wistar rat, male, (200 ± 20) g	CCI	6/8	3 days postoperatively	Treadmill, 5 days acclimatization, 16 m/min, 30 min/d, 5 d/week for 3 weeks of exercise	Mechanical pain threshold, Hot pain threshold
**(C) SNTR model**						
Cobianchi et al. (23), 2013	Sprague–Dawley rat, female, (240 + 30) g	SNTR	8/15	3 days postoperatively	Treadmill, no slope, starting speed 6 m/min, increasing by 1.2–19.2 m/min every 5 min. No further increase in velocity, 1 h/d, total of 5 d	Mechanical pain threshold, Hot pain threshold

### 3.3 The results of the risk of bias assessment

The risk of bias within the included studies was assessed using established criteria, and the evaluation results are presented in Table 2. This assessment focused on key areas such as random sequence generation, allocation concealment, blinding of outcome assessors, and selective outcome reporting. All studies adhered to complete data reporting standards. Baseline characteristics of the animals, including body weight, pre-surgery mechanical and thermal pain thresholds, were reported in all included articles. Three studies employed blinding for researchers (investigators conducting the experiment) (18, 22, 26), while six studies utilized blinding for pain testers who evaluated the animals’ responses (22, 23, 25–28).

**Table 2:** SYRCLE’s risk of bias tool for animal studies.

First author, year of publication	1	2	3	4	5	6	7	8	9	10
Huang et al. (25), 2017	U	Y	U	U	N	U	Y	N	U	Y
Tsai et al. (16), 2017	U	Y	U	U	N	U	U	N	U	Y
Kami et al. (27), 2016	U	Y	U	U	N	U	Y	N	U	Y
Almeida et al. (17), 2015	U	Y	U	U	N	U	U	N	U	Y
Chen et al. (22), 2012	U	Y	U	U	Y	U	Y	N	U	Y
Hung et al. (26), 2016	U	Y	U	U	Y	U	Y	N	U	Y
Cobianchi et al. (23), 2013	U	Y	U	U	N	U	Y	N	U	Y
Kami et al. (28), 2018	U	Y	U	U	N	U	Y	N	U	Y
Cobianchi et al. (18), 2010	U	Y	U	U	Y	U	U	N	U	Y
Kuphal et al. (29), 2007	U	Y	U	U	N	U	U	N	U	Y
Farzad et al. (24), 2018	U	Y	U	U	N	U	U	N	U	Y
Safakhah et al. (30), 2017	U	Y	U	U	N	U	U	N	U	Y

“Y” indicates a low risk of bias, “N” indicates a high risk of bias, and “U” indicates unclear risk of bias. The table evaluates ten key areas: (1) sequence generation (randomization of allocation to groups), (2) baseline characteristics (similarity of groups at the study start), (3) allocation concealment (blinding those assigning animals to groups), (4) animal placement randomization (avoiding bias in housing), (5) blinding of researchers, (6) blinding of outcome assessors, (7) blinding of testers conducting pain assessments, (8) completeness of data reporting, (9) selective outcome reporting (reporting all relevant results), and (10) other potential sources of bias.

### 3.4 Meta-analysis results

#### 3.4.1 The impact of exercise on mechanical pain threshold

**Figure 3:**
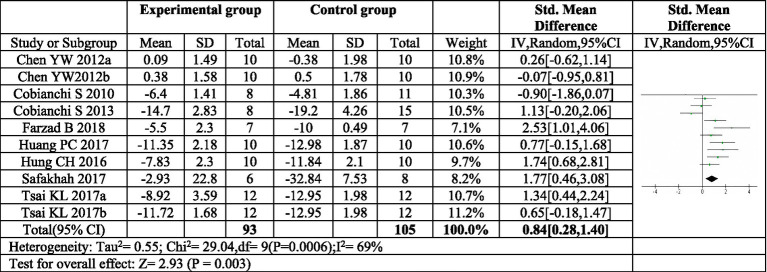
Forest plot of meta-analysis results for exercise on mechanical paw withdrawal threshold. The mechanical pain threshold of the exercise group was significantly higher than that of the control group (*p* = 0.003). ^a,b^Represent different exercise programs in the same study.

Eight articles were included in this meta-analysis (16, 18, 22–26, 30). Two of these articles investigated two distinct exercise programs, resulting in a total of ten exercise protocols evaluated (16, 22). The meta-analysis revealed a significant combined effect size (*SMD* = 0.84, 95%*CI*: 0.28–1.40, *p* = 0.003) for exercise compared to control in reducing mechanical pain sensitivity following sciatic nerve injury, as depicted in Figure 3.

#### 3.4.2 Subgroup analysis results of exercise’s impact on mechanical pain threshold

**Figure 4:**
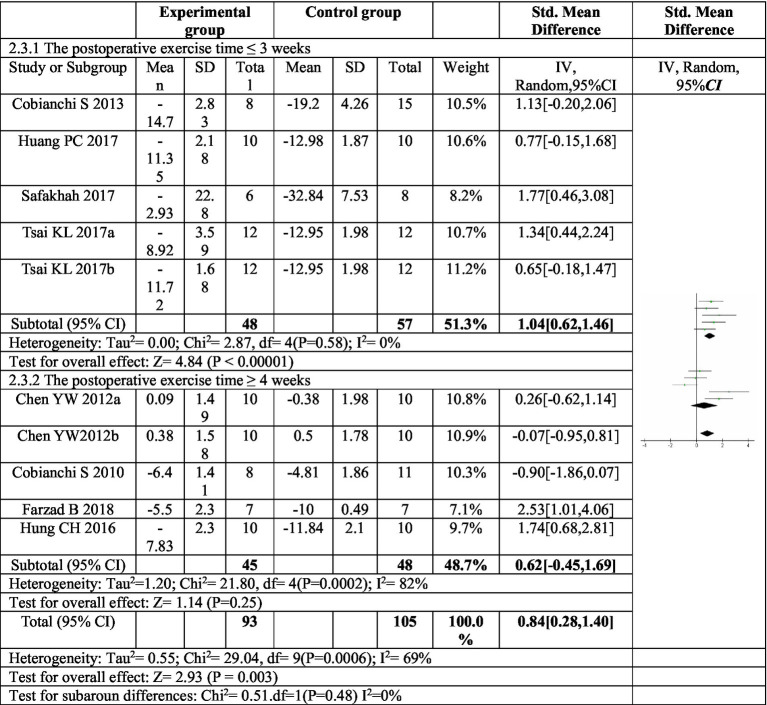
Forest plot of subgroup analysis: postoperative exercise duration on mechanical paw withdrawal threshold. Subgroup analysis based on postoperative exercise intervention time showed that postoperative exercise time ÿ 3 weeks could significantly improve the mechanical pain threshold, while postoperative exercise time ÿ 4 weeks had no significant improvement in mechanical pain threshold. ^a,b^Represent different exercise programs in the same study.

To explore potential sources of heterogeneity in the effect of exercise on mechanical pain, subgroup analyses were conducted based on exercise duration and mode (Figure 4). The analysis focused on the duration of postoperative exercise intervention. When the intervention lasted for three weeks or less (≤3 weeks), a significant increase in the mechanical pain threshold was observed in the exercise group compared to the control group (*SMD* = −1.04, 95% CI: −1.46 to −0.62, *p* < 0.00001). This finding suggests a substantial improvement in pain sensitivity with shorter exercise interventions. Importantly, the heterogeneity within this subgroup was low (*I^2^* = 0%), indicating consistency across studies. However, for exercise interventions lasting four weeks or longer (≥4 weeks), the increase in mechanical pain threshold was not statistically significant (*SMD* = 0.62, 95% *CI*: −0.45 to 1.69, *p* = 0.25). Additionally, the heterogeneity within this subgroup was high (*I^2^* = 82%), suggesting high risk of heterogeneity across studies. These results imply that exercise interventions exceeding four weeks may not be effective in improving mechanical pain sensitivity.

**Figure 5:**
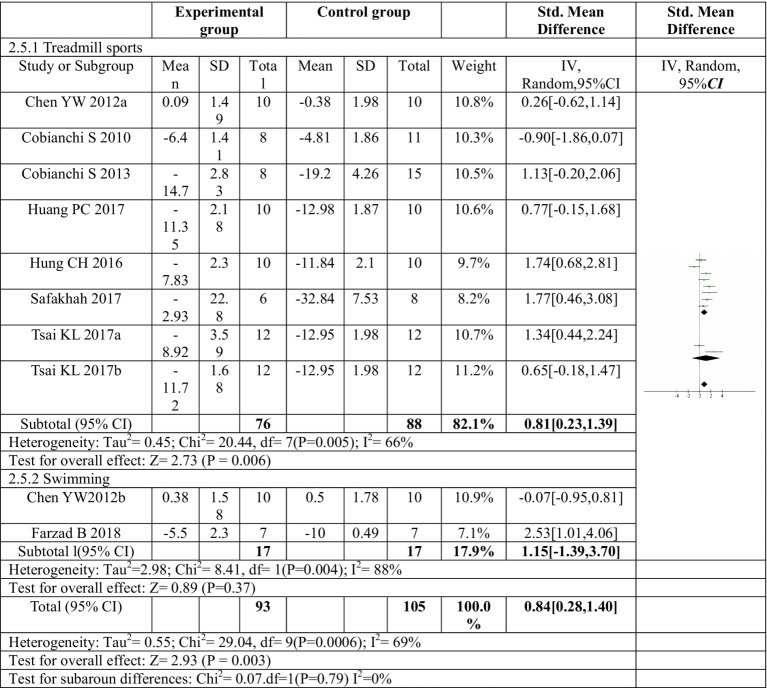
Forest plot regarding subgroup analysis of exercise forms of mechanical paw withdrawal threshold. Subgroup analysis was conducted based on different exercise methods. Treadmill exercise significantly increased the mechanical pain threshold, while swimming exercise did not significantly increase the mechanical pain threshold. ^a,b^Represent different exercise programs in the same study.

The subgroup analysis investigated the effect of different exercise modes on mechanical pain threshold, as depicted in Figure 5. Treadmill exercise showed a significant increase in mechanical pain threshold compared to the control group. The heterogeneity within the treadmill exercise group was moderate (*I*^2^ = 66%). In contrast, swimming exercise did not yield a statistically significant improvement in mechanical pain threshold. Additionally, the heterogeneity within the swimming exercise group was high (*I^2^* = 88%). These findings suggest that treadmill exercise may be more effective than swimming exercise in improving mechanical pain sensitivity following sciatic nerve injury. While swimming showed a trend toward improvement, it did not reach statistical significance in this analysis.

#### 3.4.3 Effect of exercise on thermal pain threshold

**Figure 6:**
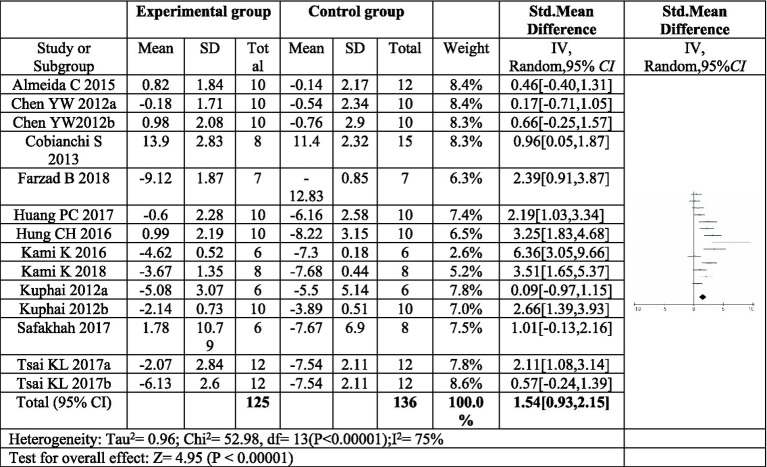
Forest plot regarding meta-analysis estimate for exercise on paw withdrawal thermal latency. The thermal pain threshold of the exercise group was significantly higher than that of the control group (*P* < 0.00001). ^a,b^Represent different exercise programs in the same study.

11 articles (16, 17, 22–30) investigated the effect of exercise on thermal pain threshold. Three of these articles included two distinct exercise programs, resulting in a total of fourteen exercise protocols evaluated (16, 20, 22). The meta-analysis revealed significant heterogeneity among the studies, necessitating the use of a random effects model. The pooled analysis indicated a significant effect size (*SMD* = 1.54, 95% CI: 0.93–2.15, *p* < 0.0001) favoring exercise compared to control in reducing thermal pain sensitivity following sciatic nerve injury (Figure 6). These findings suggest that exercise interventions may be beneficial for improving thermal allodynia in this population.

Given the limited research on exercise interventions for thermal pain and the substantial heterogeneity observed among studies, we hypothesized that variations in factors such as animal species, pain model, exercise type, timing of exercise initiation, and intervention duration might contribute to this heterogeneity. To explore these potential sources, a meta-regression analysis was conducted on the aforementioned variables (Table 3). The analysis revealed that species and pain models were not significant contributors to heterogeneity. However, the timing of postoperative exercise intervention emerged as a significant source (*p*-value = 0.037), suggesting that the time point at which exercise begins after surgery may influence treatment effects.

**Table 3:** Meta-regression analysis results of heterogeneity factors affecting paw withdrawal thermal latency.

Item	Regression coefficient	Standard error	*t*-value	*P*-value	95% *CI*
Species	−2.424	1.069	−2.270	0.053	[−4.889, 0.040]
Pain mode	−0.785	0.610	−1.290	0.234	[−2.191, 1.263]
Exercise mode	−0.519	0.773	−0.670	0.520	[−2.302, 1.263]
Start time	−0.531	0.836	−0.630	0.544	[−2.460, 1.398]
Intervention time	−2.136	0.854	−2.500	0.037	[−4.104, −0.168]

A significance level of *p* < 0.05 suggests that this factor influences the heterogeneity observed between studies.

#### 3.4.4 Subgroup analysis of the effect of exercise on thermal pain threshold

To further explore the sources of heterogeneity, a subgroup analysis was conducted based on the duration of postoperative exercise intervention. For interventions lasting four weeks or less (≤4 weeks), a significant increase in thermal pain threshold was observed in the exercise group compared to the control group (*SMD* = 1.93, 95%*CI*: 1.19–2.87, *p* < 0.00001). However, the heterogeneity within this subgroup remained high (*I^2^* = 75%).

**Figure 7:**
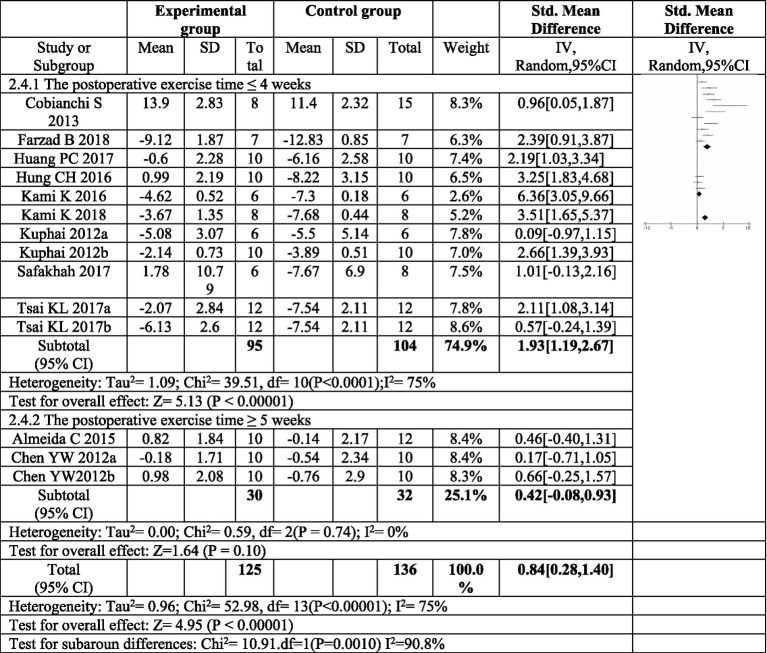
Forest plot regarding subgroup analysis of postoperative exercise duration of paw withdrawal thermal latency. In subgroup analysis with postoperative exercise time, the postoperative exercise time ≤ 4 weeks significantly increased the thermal pain threshold, and the postoperative exercise ≥ 5 weeks. There was no significant increase in the heat pain threshold. ^a,b^Represent different exercise regimens for the same study.

In contrast, exercise interventions lasting at least five weeks (≥5 weeks) showed an increase in thermal pain threshold, but this increase was not statistically significant (SMD = 0.42, 95% *CI*: 0.08–0.83, *p* = 0.10). Moreover, the heterogeneity within this subgroup was low (*I^2^* = 0%), indicating consistency in treatment effects across studies. These findings suggest that exercise interventions exceeding four weeks may not be as effective in improving thermal pain sensitivity following sciatic nerve injury, as depicted in Figure 7.

#### 3.4.5 Sensitivity analysis results

To assess the robustness of the meta-analysis findings, a sensitivity analysis was conducted. This analysis evaluated the impact of excluding individual studies on the overall effect sizes for both mechanical and thermal pain thresholds. Each study was systematically removed from the analysis one at a time, and the remaining studies were reanalyzed. The results of the sensitivity analysis revealed that the exclusion of any single study did not significantly alter the combined effect sizes for exercise on either mechanical or thermal pain thresholds. This finding suggests a high level of consistency and reliability in the overall research findings.

### 3.5 Publication bias analysis

**Figure 8:**
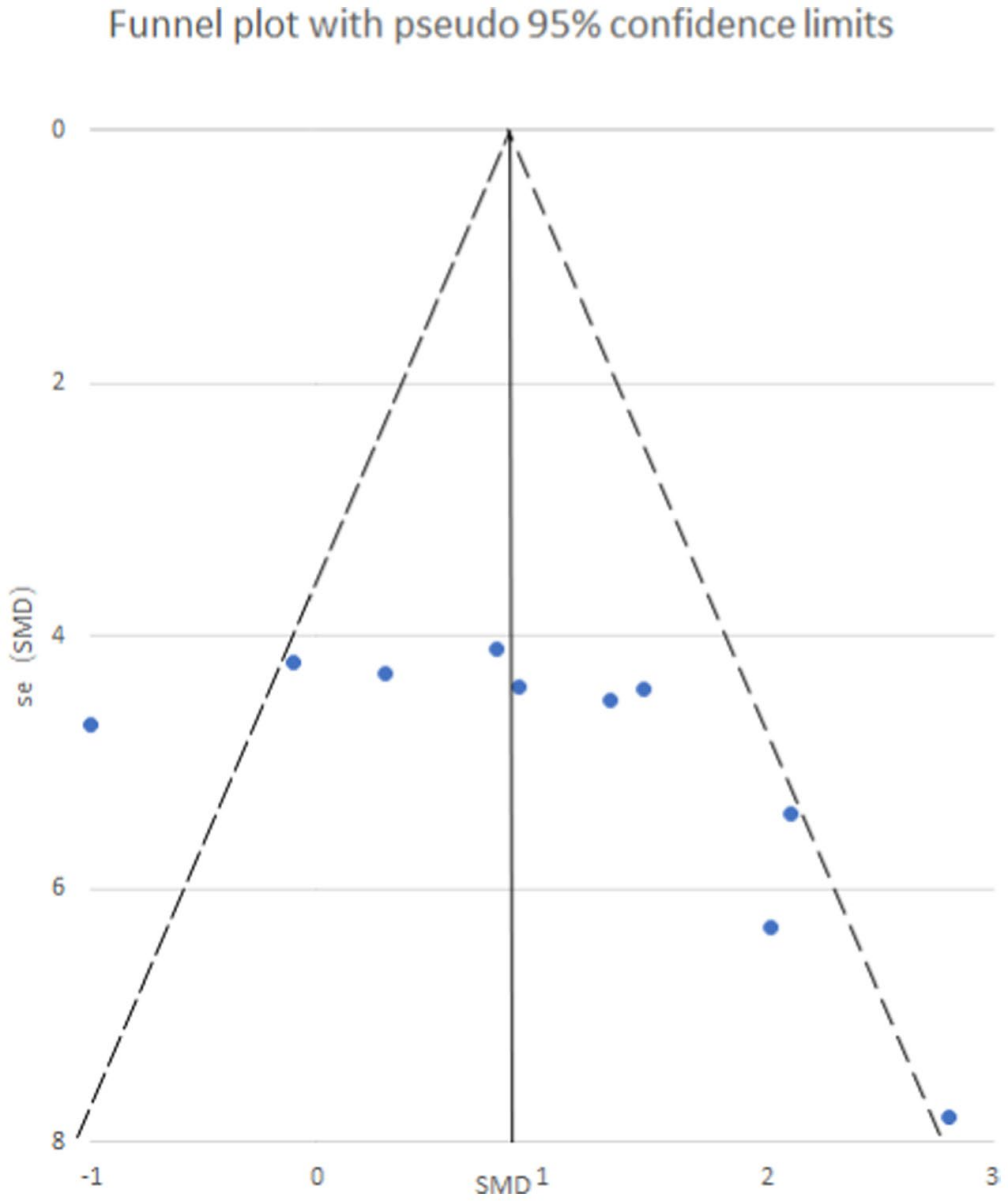
Mechanical pain threshold funnel plot. The funnel plot is symmetrical and has no publication bias.

STATA 12.0 software was employed to assess publication bias. Funnel plots were generated to visualize potential bias for both mechanical and thermal pain thresholds following exercise intervention (Figure 8). Additionally, Egger’s regression test was utilized for quantitative analysis of publication bias.

**Figure 9:**
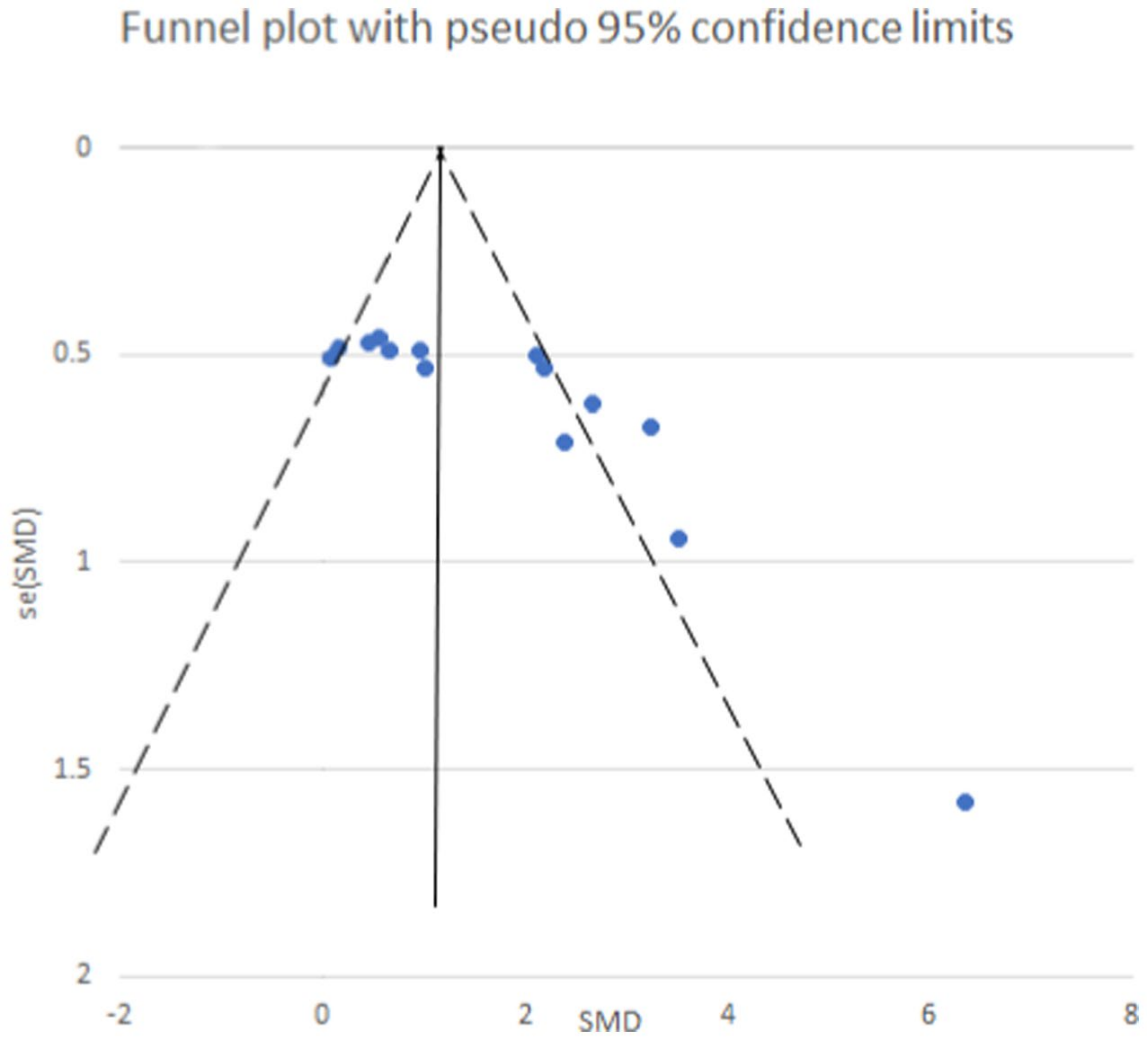
Thermal pain threshold funnel plot. The funnel plot is asymmetrical and there is publication bias.

**Figure 10:**
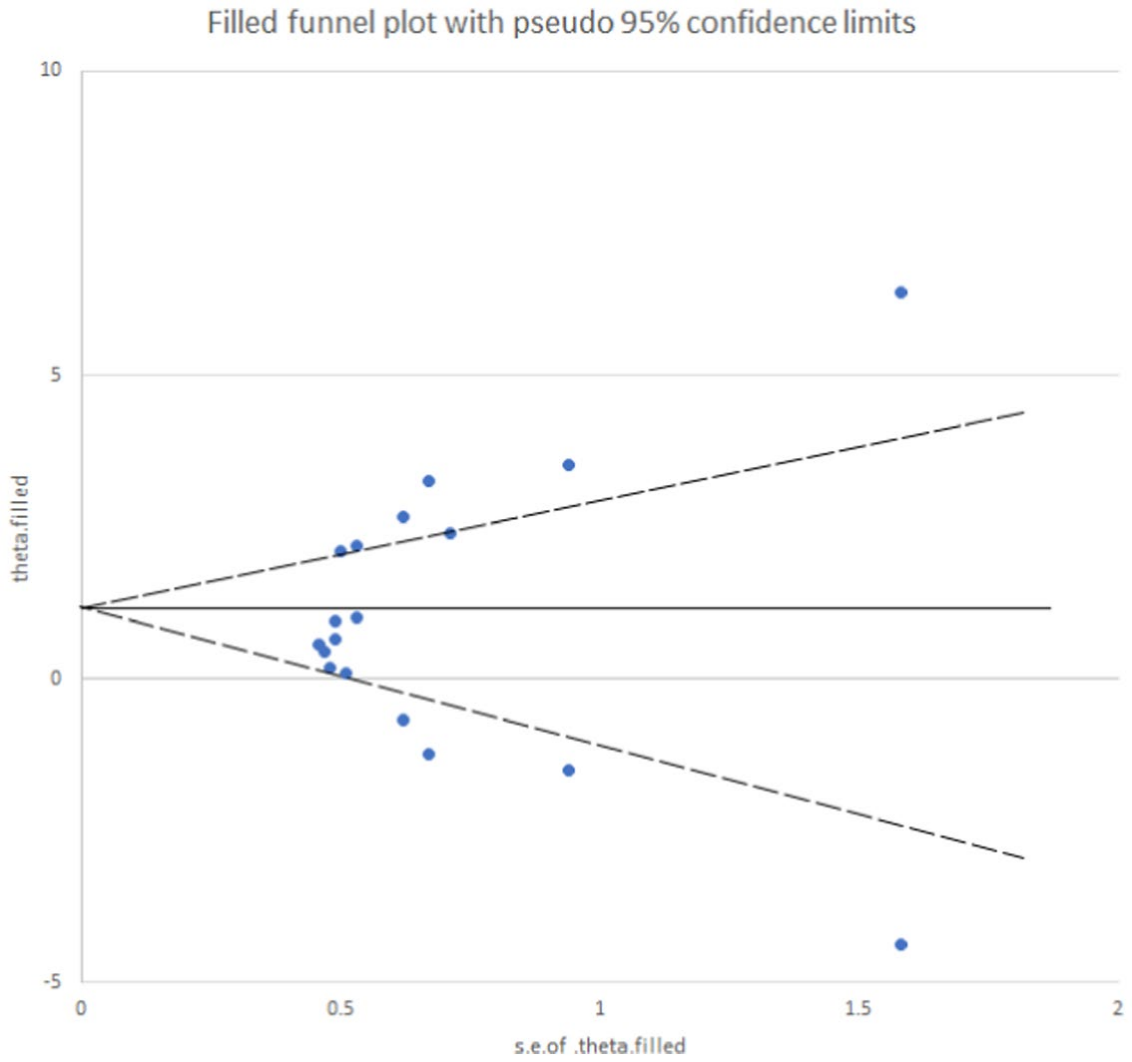
Analysis chart of trim and fill method for thermal pain threshold index. After adding four studies using the trimming and patching method, publication bias was eliminated.

Egger’s test yielded a *p*-value of 0.073, suggesting an absence of publication bias for the analysis of mechanical pain threshold. Conversely, the funnel plot for thermal pain threshold (Figure 9) exhibited asymmetry, and Egger’s test confirmed this with a highly significant p-value of less than 0.001, indicating the presence of publication bias. To address this bias, a trim and fill method was implemented (Figure 10). This method statistically imputes missing studies to achieve a symmetrical funnel plot, suggesting the addition of four hypothetical studies. Despite a slight change in the effect size after adjusting for publication bias (*SMD* = 2.81, 95%*CI*: 0.31–1.74, *p* = 0.005), a significant difference between the exercise and control groups remained.

Thermal pain perception involves not only TRPV1 channels, but may also be affected by other temperature-sensitive ion channels, neurotransmitters, and receptors. In addition, thermal stimulation may trigger a wider range of neuroinflammatory responses, which may show greater variability under different experimental conditions. Mechanical pain perception is relatively more direct, and its neural pathways may be relatively simple. Therefore, under experimental conditions, changes in mechanical pain thresholds may be more consistent and predictable. The reasons why thermal responses may show deviations in meta-analysis involve many factors, such as the complexity of pain mechanisms, differences in experimental methods, uncertainties in data collection and analysis, and physiological adaptation and fatigue effects. These factors work together to lead to inconsistencies in the results of thermal pain thresholds in different studies. However, by adopting appropriate statistical methods and interpretation strategies, we can reduce this bias to a certain extent and improve the reliability and validity of meta-analysis results.

## 4 Discussion

### 4.1 The effect of postoperative exercise intervention duration on mechanical pain threshold

Subgroup analysis revealed that exercise interventions lasting four weeks or less (≤4 weeks) significantly improved mechanical pain sensitivity compared to control, while interventions exceeding four weeks (≥ 4 weeks) did not. This finding aligns with a previous meta-analysis by Guo et al. (14) on exercise for peripheral neuropathy in rats, which showed no significant improvement in mechanical pain sensitivity with interventions lasting five weeks or longer. Similarly, Chen et al. (22) investigated the effect of exercise on mechanical pain threshold using a chronic compression sciatic nerve injury model in rats. Their findings showed a significant difference in pain threshold between exercise and control groups early after surgery, but no difference by day 39 post-surgery. This suggests minimal improvement after six weeks of exercise. Grace et al. (31) also used a chronic compression model and found that mechanical pain sensitivity persisted in the control group for 15 weeks. Variations in pain duration across similar models might be due to differences in the severity of nerve damage caused by the specific pain model, potentially contributing to the observed heterogeneity in our findings. Overall, the duration of the exercise intervention significantly impacted the collective results. Most studies demonstrated a positive effect on reducing mechanical pain sensitivity before the end of the exercise program. These findings suggest that the duration of postoperative exercise plays a critical role in improving mechanical pain sensitivity through physical activity. Notably, exercise interventions within three weeks of surgery appear to have the most pronounced impact on improving mechanical pain sensitivity following sciatic nerve injury.

### 4.2 The effect of postoperative exercise intervention duration on thermal pain threshold

Sciatic nerve injury is known to significantly reduce thermal pain threshold and lead to thermal sensitivity symptoms. Assessing thermal pain threshold involves measuring the latency in response to thermal radiation. However, inconsistencies in the literature regarding the intensity of thermal radiation used during experiments can lead to varying outcomes. This variation in methodology for thermal pain threshold testing may be a significant contributor to the observed heterogeneity in results across studies.

A comprehensive review by Pitcher et al. (19) examined the impact of exercise on pain models in mice. Their analysis included 22 studies investigating thermal pain sensitivity in exercise-treated pain models for both rats and mice. Notably, the majority of these studies reported significant improvement in thermal pain sensitivity following exercise intervention. Pitcher et al. considered several factors that might influence the effectiveness of exercise, including animal characteristics (gender and species), timing and type of exercise initiation, the specific pain model used, and the duration of the exercise program (19). Importantly, their review did not identify a single factor that definitively influenced the success of exercise in enhancing thermal sensitivity. However, their findings echo the results of the current study. Exercise interventions lasting four weeks or less (≤4 weeks) were associated with a significant improvement in thermal pain sensitivity following sciatic nerve injury. In contrast, exercise programs exceeding five weeks (≥5 weeks) did not yield a significant improvement in thermal pain sensitivity, but may still play a role in promoting recovery processes.

### 4.3 Analysis of the reasons for the differences in the results of postoperative exercise intervention on mechanical pain threshold and thermal pain threshold

Mechanical and thermal pain are mediated by distinct receptors and neural pathways. At the primary level of sensory input, these pathways differentiate between various physical sensations, including nociceptive heat/cold, mechanical stimulation, and itching (32). Studies have shown that Mas-related G protein-coupled receptors (MRGPRs) are prominently expressed in primary sensory neurons (33). Specifically, nociceptors expressing transient receptor potential vanillic acid receptor 1 (TRPV1) are responsible for thermal pain, while mechanical pain is mediated solely by nociceptors (34). Importantly, there is no observed interaction between these two receptor systems in pain production (35). Furthermore, nerve growth factor (NGF) plays a crucial role in neuropathic pain development (36). Its high-affinity receptor, tyrosine kinase receptor A (TrkA), mediates mechanical pain sensitivity resulting from cutting injury and inflammatory pain, but does not affect basal mechanical and thermal pain thresholds (37). These findings, along with the influence of higher neural centers on pain processing, highlight the distinct characteristics of mechanical and thermal pain generation. Consequently, the variations in underlying mechanisms contribute to the observed inconsistencies in the effects of exercise interventions on these two types of pain (38).

### 4.4 Analysis under different pain models

When exploring exercise as an effective means of relieving pain from sciatic nerve injury, we must not only focus on the overall conclusion of the impact of exercise duration on pain behavior, but also need to conduct an in-depth analysis of the development, duration, and spontaneous recovery of hypersensitivity reactions under different pain models. Characteristic. These subtle differences are critical to fully understanding the effects of exercise interventions and developing targeted pain management strategies. The following is a detailed analysis and discussion of this issue.

This meta-analysis shows that in animal models of sciatic nerve injury, short-term postoperative (≤3 weeks) exercise intervention significantly improves mechanical pain thresholds, while mid-term postoperative (≤4 weeks) exercise intervention significantly improves mechanical pain thresholds. Thermal pain threshold. However, when the duration of exercise was extended to more than 4 weeks, its relief effect on mechanical pain and thermal pain was no longer significant. This finding suggests that exercise can effectively reduce pain hypersensitivity caused by nerve damage within a specific period of time, but beyond this time window, its effect may gradually weaken or disappear.

#### 4.4.1 Differences in specificity among different pain models

Although the overall trends are consistent, different pain models exhibit significant differences in the development and duration of hypersensitivity reactions and spontaneous recovery. These differences may arise from a variety of factors, including the severity of the nerve injury, the anatomy of the injury site, and the physiological responses of the individual animals (39).

#### 4.4.2 Development and duration of hypersensitivity reactions

The rate of development and duration of hypersensitivity reactions vary in different pain models. For example, in some models, significant mechanical and thermal pain hypersensitivity occurs immediately after nerve injury and lasts from weeks to months; in other models, the hypersensitivity may be more gradual and persistent. The time is relatively short. This difference directly affects the optimal time point and duration of exercise intervention (40).

#### 4.4.3 Spontaneous recovery phenomenon

Of note, animals in some models experience spontaneous recovery of hypersensitivity reactions within weeks of injury (41). This phenomenon shows that the nervous system has a certain self-healing ability and can reduce pain hypersensitivity to a certain extent. Therefore, long-term exercise intervention may not provide additional pain relief in these models because the animals are already in the process of spontaneous recovery. However, this does not mean that exercise intervention has no value in alleviating initial hypersensitivity reactions; on the contrary, short-term exercise intervention can still significantly reduce pain levels and provide positive support for the animal’s recovery process (42).

#### 4.4.4 Deep explanation and recognition of exercise effectiveness

##### 4.4.4.1 Discussion on physiological mechanisms

The reason why exercise can alleviate the pain hypersensitivity caused by sciatic nerve injury to a certain extent may be related to its impact on multiple physiological mechanisms. Exercise can promote blood circulation, increase the release of neurotrophic factors, and promote nerve regeneration and repair (43). At the same time, exercise can also regulate the function of the immune system, reduce inflammatory reactions and neuroimmune reactions, thereby indirectly relieving pain (44). However, the effects of these physiological mechanisms may be affected by factors such as pain model specificity and exercise duration.

##### 4.4.4.2 Recognize the importance of model specificity

When drawing conclusions about the effectiveness of exercise, we must acknowledge differences in specificity between pain models. This difference is not only reflected in the development and duration of hypersensitivity reactions, but may also affect the specific effects and application strategies of exercise intervention. Therefore, when formulating an exercise-based pain management program, we need to fully consider factors such as individual pain model characteristics (45, 46), injury severity, and physiological responses to achieve personalized and precise treatment (47).

#### 4.4.5 Suggestions for future research directions

In order to further verify and expand the conclusions of this article, future research can start from the following aspects:

Refining the classification of pain models: Carry out a more detailed classification and comparison of different sciatic nerve injury models to clarify their specific differences in the development, duration and spontaneous recovery of hypersensitivity reactions.
Explore the optimal time window for exercise intervention: Through more experimental studies, determine the optimal starting time and duration of exercise intervention under different pain models to maximize its pain relief effect.
In-depth study of the impact of exercise on physiological mechanisms: using advanced molecular biology and neuroimaging techniques, in-depth exploration of the specific impact of exercise intervention on physiological mechanisms such as blood circulation, neurotrophic factor release, immune system function, and its relationship with pain relief internal connections.
Formulation of personalized treatment plans: Develop personalized exercise treatment plans based on individual pain model characteristics, physiological responses and other factors to improve the pertinence and effectiveness of pain management.

### 4.5 Reasons for the few effects of long-term exercise

#### 4.5.1 Spontaneous recovery phenomenon

It is pointed out that in some models, untreated animals will spontaneously recover over time, and their pain thresholds gradually approach or reach the level of trained animals (48). Therefore, although prolonged exercise may help with initial relief, the later effects are no longer significant.

#### 4.5.2 Exercise fatigue or adaptation

Prolonged exercise may cause fatigue or adaptation in animals, thereby reducing the pain-relieving effect of exercise (49).

#### 4.5.3 Complexity of pain mechanisms

Emphasizes the complexity of pain mechanisms, which may involve changes in multiple neurotransmitters, receptors, and ion channels (50). Prolonged exercise may not be sufficient to sustainably affect these complex pathophysiological processes.

## 5 Limitations of this research

This study has several limitations that should be acknowledged. Incomplete data and the inability to contact original authors for clarification resulted in the exclusion of potentially relevant studies. Additionally, variations in exercise interventions and pain measurement methods across studies may have contributed to the observed inconsistencies in results. Despite employing sensitivity and subgroup analyses, the issue of heterogeneity remains unresolved. Furthermore, the limited literature available on exercise interventions for chronic pain primarily involves male subjects. This restricts our ability to draw definitive conclusions regarding potential gender-based differences in the analgesic efficacy of exercise. Inconsistencies in the types of exercise employed further complicate efforts to compare exercise intensity and elucidate the relationship between intensity and pain reduction (24, 25). While exercise remains a cornerstone of pain management, tailored to individual presentations, further research is necessary to address these limitations. Given established sex differences in pain mechanisms (51), future studies should explore whether exercise has differential effects on pain improvement based on gender.

## 6 Conclusion

Exercise has emerged as an effective intervention for improving both mechanical and thermal allodynia resulting from sciatic nerve injury in rodents. Notably, the species, method, and timing of exercise initiation appear to have minimal influence on the overall effectiveness of the intervention. However, the duration of pain sensitization in the specific pain model significantly impacts the success of exercise-induced analgesia. Studies have shown that exercise demonstrably improves sciatic nerve damage in animals. Postoperative exercise interventions lasting less than three weeks resulted in a significant improvement in mechanical allodynia, while those lasting less than four weeks yielded a significant improvement in thermal hyperalgesia. These findings highlight the importance of considering the duration of pain sensitivity within the chosen pain model for future research on exercise-induced analgesia.

Future research should prioritize investigating the specific exercise movements or modalities that induce positive changes in pain-related molecules. While exercise demonstrates promise as a pain management strategy, it is important to acknowledge that it may not be a universally effective treatment for all types of pain. Therefore, further studies are needed to explore the potential benefits of combining exercise with existing therapies such as medication, acupuncture, and even novel approaches like stem cell tissue engineering. This multi-faceted approach has the potential to address pain relief from various perspectives and improve patient outcomes.

In summary, the different responses of mechanical pain threshold and thermal pain threshold to exercise intervention may originate from their respective unique molecular mechanisms. Further studies should delve into the interplay between these mechanisms and how they jointly regulate the process of neuropathic pain. By deeply understanding these molecular features, we can provide a theoretical basis for developing more precise and effective pain treatment strategies.

## Data availability statement

The original contributions presented in the study are included in the article/supplementary material, further inquiries can be directed to the corresponding authors.

## Author contributions

SL: Formal analysis, Writing – original draft. QL: Data curation, Writing – review & editing. HW: Visualization, Writing – review & editing. HZ: Data curation, Project administration, Writing – review & editing. QZ: Project administration, Writing – review & editing. JS: Methodology, Writing – review & editing. JZ: Supervision, Writing – review & editing. PF: Funding acquisition, Supervision, Writing – review & editing. AZ: Supervision, Funding acquisition, Writing – original draft, Writing – review & editing.
